# A systematic review of causal pathways of socioeconomic inequalities in stroke

**DOI:** 10.1177/17474930251399064

**Published:** 2025-11-12

**Authors:** Camila Pantoja-Ruiz, Lu Liu, Evelyn Lim, Marina Soley-Bori, Wasana Kalansooriya, Eva Emmett, Abdel Douiri, Yanzhong Wang, Ajay Bhalla, Amal R Khanolkar, Divya Parmar, Sabine Landau, Matthew DL O’Connell, C D A Wolfe, Iain J Marshall

**Affiliations:** 1 Department of Population Health Sciences, School of Life Course & Population Sciences, King’s College London, London, UK; 2 Guys and St Thomas NHS Foundation Trust, London, UK; 3 Institute of Psychiatry, Psychology & Neuroscience, King’s College London, London, UK

**Keywords:** Socioeconomic status, inequalities, public health, hypertension, rehabilitation, stroke risk

## Abstract

**Background:**

Socioeconomic status (SES), often measured by education, income, occupation, or area-level deprivation, impacts stroke incidence and outcomes, yet the underlying mechanisms remain unclear. This review synthesizes causal analyses quantifying drivers of these inequalities.

**Methods:**

We conducted a systematic review (PROSPERO CRD42024554285) and reported following the PRISMA-2020 guidelines. Observational studies applying causal mediation analysis between SES and stroke risk, disability, or mortality were included from PubMed, Embase, Scopus, and Google Scholar. SES indicators, outcomes, mediators, and decompositions into natural direct effect (NDE) and natural indirect effect (NIE) were extracted. Risk of bias and certainty of evidence were assessed using ROBINS-E and GRADE. A narrative synthesis was undertaken, and findings were illustrated in causal diagrams.

**Results:**

Of 12,034 records, 19 studies (15 in high-income countries) were included. Lower SES increased stroke incidence through hypertension (NIE 14–21% of the total effect, moderate certainty), although one study restricted to women reported smaller effects (2–4%). Smoking (6–19.9%, very low certainty). At 3 months post-stroke, the combined outcome of death or disability was higher due to severe strokes (38.5% for ischemic, 57–94% for hemorrhagic, moderate certainty). One study found that hypertension, atrial fibrillation, and smoking together mediated 28.5% of the SES effect on stroke severity (low certainty). Reduced access to thrombolysis and stroke units mediated 2.7% of 3-month disability/mortality (very low certainty), while greater distance to specialized centers explained 48% of inequalities in thrombectomy access (low certainty). Long-term mortality (⩾6 months) was mediated by comorbidities (18%) and healthcare coverage (24–55%), both with low certainty.

**Conclusions:**

Hypertension, smoking, and differential stroke severity at presentation are the main pathways through which low SES increases stroke risk and causes worse outcomes. Targeting these may reduce inequalities, though evidence from low-income settings and emerging mediators (e.g. early-life SES, environmental exposures, care quality) is lacking.

## Graphical abstract

**Graphical abstract:**
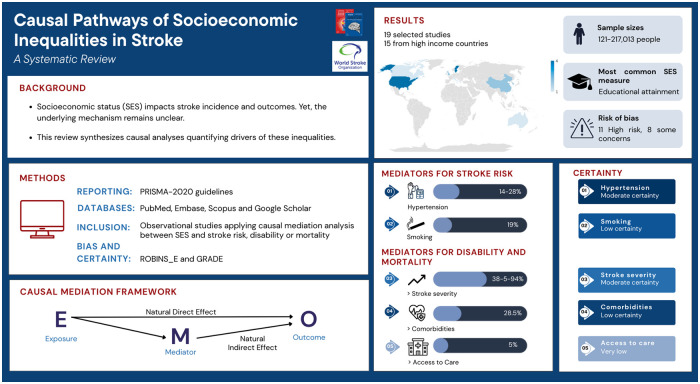

## Introduction

Stroke remains a major global health challenge, with over 12 million new cases annually, accounting for 10.7% of deaths and 5.6% of disability-adjusted life years (DALYs) worldwide.^1^ People with lower socioeconomic status (SES) experience higher stroke risk, greater disability, and increased mortality.^2,3^ Inequalities are most pronounced in low- and middle-income countries (LMICs), where elevated cardiovascular risk factors (e.g. hypertension, diabetes, dyslipidemia, smoking, physical inactivity, poor diet, and obesity), environmental exposures (e.g. air quality), and limited access to care intensify stroke burden.^2,4^ However, inequalities also persist in high-income countries (HICs), where lower SES groups present with more severe strokes and reduced access to reperfusion therapies and stroke units.^5–9^

SES is a multidimensional construct reflecting access to resources and social standing, commonly measured by education level, income, or occupation.^10,11^ Traditional epidemiological studies consistently link lower SES with higher stroke incidence and poorer outcomes.^8,12^ While these studies suggest pathways, such as vascular risk, health behaviors, or care access, through which SES may act, they cannot quantify the contribution of each mediator or establish causality.^13,14^

To address this, recent studies increasingly use causal inference methods to estimate the extent to which SES effects operate through specific mediators.^13,14^ These methods allow more robust estimation under explicit assumptions, yet they remain sensitive to residual confounding and measurement error in SES and mediator variables.^13,14^

## Aim

This systematic review synthesizes evidence from studies using causal mediation analysis to examine and quantify how SES affects stroke risk, disability, and mortality. By synthesizing quantitative evidence on pathways driving stroke inequalities, we aim to identify actionable targets to inform policies and clinical strategies with the potential of reducing these inequalities.

## Methods

This review was registered in PROSPERO (ID-CRD42024554285) and follows PRISMA-2020 guidelines.

### Eligibility criteria

We included observational studies (cohort, case–control, or cross-sectional) applying causal mediation methods to examine pathways between SES (education, income, occupation, or area-based indicators) and stroke risk, disability, or mortality. Studies had to hypothesize and test at least one mediator.

For cohort studies, stroke incidence was defined as first-ever events using the WHO criteria.^15^ Case–control and cross-sectional studies were eligible if they examined the odds or prevalence of physician-diagnosed stroke. Disability was measured by the modified Rankin scale (mRS) or Barthel-Index^16,17^ and mortality through case fatality at any time post-stroke.^18^ Studies assessing stroke severity or care as mediators of disability or mortality were eligible. We excluded qualitative designs, associative-only analyses, or those without stratified SES data (Panel 1).

### Information sources and search strategy

Searches were conducted in PubMed, Embase, Scopus, and Google Scholar (initial: 5 June 2024; update: 25 April 2025). Strategy combined terms for SES, stroke, and mediation analysis (Supplemental Panels 1 and 2). Reference lists of included studies and reviews were hand-searched for additional records.

### Mediation analysis framework

We applied VanderWeele’s causal mediation framework, decomposing the total effect (TE) of SES into a natural direct effect (NDE); the portion not operating through a mediator and a natural indirect effect (NIE); and the portion transmitted via a mediator.^14^ Mediators are defined as variables hypothesized to transmit part of the SES effect on stroke risk or outcomes.^14^ For continuous outcomes, TE equals the sum of NDE and NIE. The mediated proportion is calculated as NIE/TE. Despite simplifying the multidimensional nature of SES, this enables structured comparisons of mediation across studies and is well established in public health and social epidemiology.^19–21^

### Study selection and data collection

Three reviewers (C.P.-R., L.L., and E.E.) screened titles/abstracts using Rayyan.ai,^22^ disagreements were resolved by W.K. Full texts were assessed by C.P.-R. and I.J.M. Data extraction was performed by C.P.-R. and independently checked by E.L. Extracted items included study characteristics, SES measures, mediators, methods, and effect estimates.

### Risk of bias assessment

Study quality was assessed using the Risk of Bias in Non-randomized Studies of Exposures (ROBINS-E) tool,^23^ considering bias due to confounding, exposure/outcome measurement, participant selection, post-exposure interventions, missing data, and selective reporting. Assessments were done by C.P.-R., reviewed by E.L., and discrepancies resolved by I.J.M.

### Synthesis of results

Due to heterogeneity in SES measures, outcome measures, mediators, and analytical approaches, narrative synthesis following Popay et al.’s^24^ approach was conducted. All findings were extracted and reported in the tables and the Results section; only significant indirect effects (95% CI not crossing zero or p < 0.05) are visualized in causal diagrams, iteratively developed by CPR, IJM, and co-authors. Findings were grouped by outcome into stroke risk and post-stroke disability/mortality.

Certainty of evidence for each pathway was rated with GRADE.^25^ Starting with “low” quality for observational studies, we downgraded for risk of bias, inconsistency, indirectness, and imprecision, and upgraded for large magnitude and consistency of the effect.^25^

## Results

### Search results and study selection

**Figure 1.**
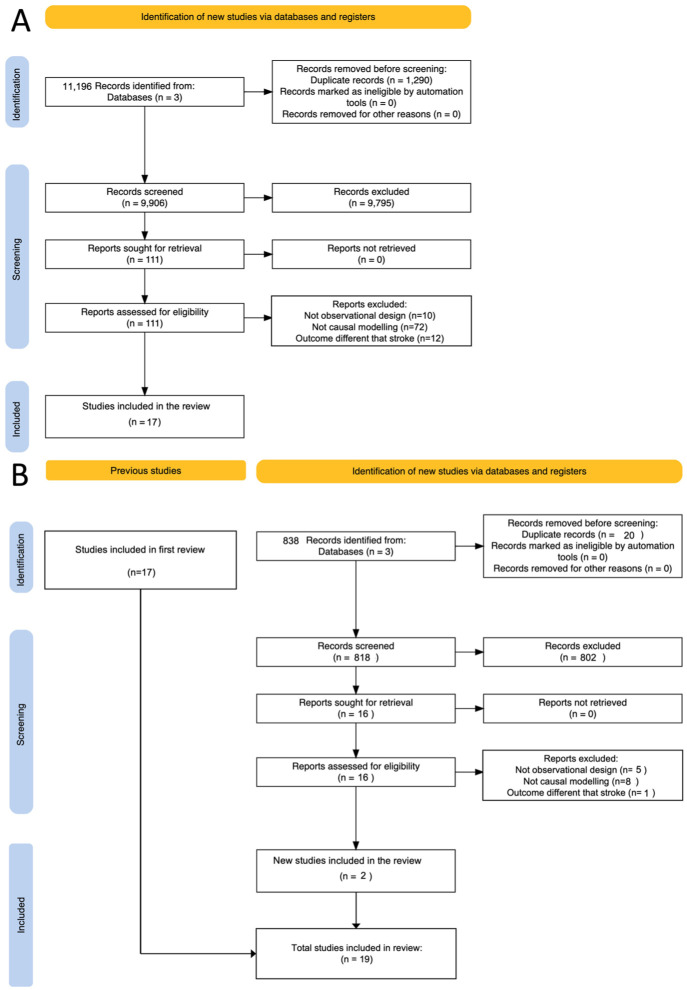
PRISMA diagram showing flow of studies in the review: (a) initial search and screening process and (b) updated search.

The initial search retrieved 11,196 records. After 1290 duplicates were removed, 9906 titles/abstracts were screened, resulting in 111 full texts assessed. Initially, 17 studies met the inclusion criteria.^26–42^ The updated search identified 838 additional records; after removing 20 duplicates, 818 were screened, with 16 assessed in full-text and 2 included.^43,44^ In total, 19 studies were included (Figure 1).

### Characteristics of included studies

The 19 studies,^26–44^ published between 2012 and 2025, comprised 13 cohort studies,^26–32,34–37,43,44^ 1 case–control,^33^ and 5 cross-sectional.^38–42^ Sample sizes ranged from 121^42^ to 217,013^26^ (Table 1 and Supplemental Table 1). Fifteen studies were from HIC^26–31,33,35–40,43,44^ and four from LMIC^32,34,41,42^ (Supplemental Figure 1).

**Table 1.** Characteristics of included studies.

Author (year)	Country	Study design	Sample size	Population	Study period	SES measure(s)	Outcome	Mediators examined	Analytical approach
**Stroke risk**									
Carter et al.^26^	UK	Cohort (prospective)	217,013	Adults aged 40–69, stroke-free at baseline (UK-Biobank)	2006–2010	Education	Incidence of first-ever stroke	SBP, BMI, smoking	Counterfactual-based causal mediation
Jackson et al.^31^	Australia	Cohort (prospective)	11,468	Mid-aged women (ALSWH, aged 47–52 at baseline)	1996–2010	Education, homeownership	Incidence of first-ever stroke	Lifestyle factors, biological factors, and depression	Change-in-estimate/regression
Jeong et al.^32^	South Korea	Cohort (prospective)	213,526	Adults ⩾ 18 years (NHIS)	2006–2015	Income	Incidence of stroke	Metabolic syndrome (BMI, cholesterol, SBP)	Counterfactual-based mediation
Lee et al.^34^	South Korea	Cross-sectional	19,147	Adults ⩾ 19 years (KNHANES survey)	2013–2016	Cumulative social risk score	Odds of physician-diagnosed stroke (self-report)	Framingham risk score components	Baron & Kenny/regression decomposition
Nandi et al.^38^	USA	Cohort (longitudinal, HRS panel)	9055	Adults born 1931–1941, stroke-free at baseline	1992–2006	Early-life SES (composite)	Self-reported physician-diagnosed stroke	Adult SES	Marginal structural models
Kastorini et al.^33^	Greece	Case–control (hospital-based)	1000	250 first-ever ischemic stroke cases, 250 stroke-free controls, 250 ACS cases, 250 ACS controls	2009–2010	Education, financial status, and occupation	Odds of first-ever ischemic stroke	Diet, physical inactivity, depression	SEM
Ricceri et al.^40^	Italy	Cohort (prospective, EPICOR)	43,791	Adults aged 35–70	1993–2008	Education	Incidence of ischemic stroke	Lifestyle and vascular risk factors	SEM
Wang et al.^44^	UK	Cohort (prospective, UK Biobank)	447,227	Adults aged 40–70	2006–2019	Education	Incidence of first-ever stroke (WHO/ICD)	Lifestyle cluster (smoking, alcohol, diet, physical inactivity, sleep), SBP, BMI, glucose, cholesterol	Counterfactual-based mediation
**Disability after stroke**									
Eagles et al.^27^	Canada	Cohort (retrospective)	1335	Ischemic stroke patients treated with alteplase	2017–2019	Area-based deprivation index	Disability (EVT access; distance to center)	Stroke center access, geographical distance	Counterfactual-based mediation (linear/logistic models)
Ghoneem et al.^29^	USA	Cohort (prospective)	1098	Acute ischemic stroke patients	2009–2011	Zip-code median income	mRS (3 months)	Infarct volume, NIHSS	Counterfactual-based regression mediation
Lindmark et al.^36^	Sweden	Cohort (retrospective)	86,316	Ischemic stroke patients	2012–2016	Education	Stroke severity	Risk factors, secondary prevention drugs	Interventional disparity effects
Lindmark et al.^43^	Sweden	Cohort (register-based)	6910	Working-age stroke patients (18–64 years)	2015–2017	Composite (education + income)	ADL (3 months)	Smoking, diabetes, hypertension, AF, stroke type, LOC, statin use	Counterfactual-based mediation
Zhang et al.^41^	China	Cohort (nested RCT)	151	Ischemic stroke patients	2015–2016	Education	Disability (6 months)	Health knowledge, beliefs	SEM
Zhang et al.^42^	China	Cross-sectional	121	Ischemic stroke patients	2018–2020	Education	Disability (6 months)	Depression, illness resources	SEM
**Post-stroke mortality**									
Lindmark et al.^37^	Sweden	Cohort (nationwide)	25,846	Ischemic stroke patients independent in ADL pre-stroke	2015–2016	Composite (education + income)	Death or ADL dependency at 3 months	Comorbidity, stroke severity, and acute care	Interventional disparity effects
Lindmark et al.^35^	Sweden	Cohort (nationwide)	57,936	First-ever stroke patients	2009–2012	Income	3 months case fatality	Stroke severity	Interventional disparity effects
Hyldgård et al.^30^	Denmark	Cohort (retrospective)	59,066	First-ever ischemic stroke patients	2003–2018	Education, family income	30-day mortality and readmission	Quality of acute stroke care	Counterfactual-based mediation (imputation)
Fan and Lam^28^	USA	Cohort (longitudinal)	17,228 PY (MI); 14,113 PY (stroke)	Adults > 50 years with stroke across three cohorts	1996–2016	Education	All-cause mortality	Comorbidities, income, occupation, and healthcare coverage	KHB decomposition
Potter et al.^39^	USA	Cohort (retrospective)	677	Intracerebral hemorrhage patients	2016–2021	Area deprivation index	Severe disability or death (mRS 4–6 at 90 days)	Stroke severity	SEM

SBP: systolic blood pressure; BMI: body mass index; SEM: structural equation modeling; ACS: acute coronary syndrome; ALSWH: Australian Longitudinal Study on Women’s Health; NHIS: National Health Insurance Service (South Korea); KNHANES: Korea National Health and Nutrition Examination Survey; HRS: Health and Retirement Study (USA); EPICOR: European Prospective Investigation into Cancer and Nutrition—Cardiovascular; mRS: modified Rankin Scale; BI: Barthel Index; ADL: activities of daily living; LOC: level of consciousness; AF: atrial fibrillation; EVT: endovascular thrombectomy; NIHSS: National Institutes of Health Stroke Scale; KHB: Karlson–Holm–Breen method; PY: person-years.

**Definitions:**

• *Incidence refers to first-ever stroke events (WHO/ICD definitions)*

• *Odds of physician-diagnosed stroke refer to self-reported doctor diagnosis in cross-sectional surveys or longitudinal self/proxy reports*

• *Disability includes post-stroke functional outcomes measured by mRS, BI, or ADL/IADL*

• *Mortality includes case-fatality, all-cause mortality, or combined death/ADL dependency, as defined in each study.*

Educational attainment was the most common SES measure (n = 12),^26,28,31,33,34,36,37,40–42,44^ followed by income (n = 6).^27,29,32,35,37,38^ Five studies used multiple indicators.^31,33,34,37,38^ Supplemental Tables 2 and 3 summarize the TE of SES on stroke risk and outcomes.

Using the ROBINS-E tool, 11 studies were rated high risk of bias and 8 had some concerns,^27–32,34–36,38,41,43,44^ mainly due to residual confounding and self-reported or area-level SES measures (Supplemental Table 4). No study was rated as low risk. Certainty was generally low to very low, with only a few pathways graded moderate (Supplemental Tables 5 and 6).

### NIE of SES on stroke risk

Eight studies examined pathways driving SES inequalities in stroke risk (including incidence in longitudinal cohorts and odds of diagnosis in case–control and cross-sectional studies) over follow-up periods ranging from 3 to 20 years.^26,31–34,38,40,44^ Methods included counterfactual-based causal mediation (n = 4),^26,32,38,44^ structural equation modeling(n = 2),^33,40^ regression (n = 1), and product-of-coefficients (n = 1).^31,34^

**Figure 2.**
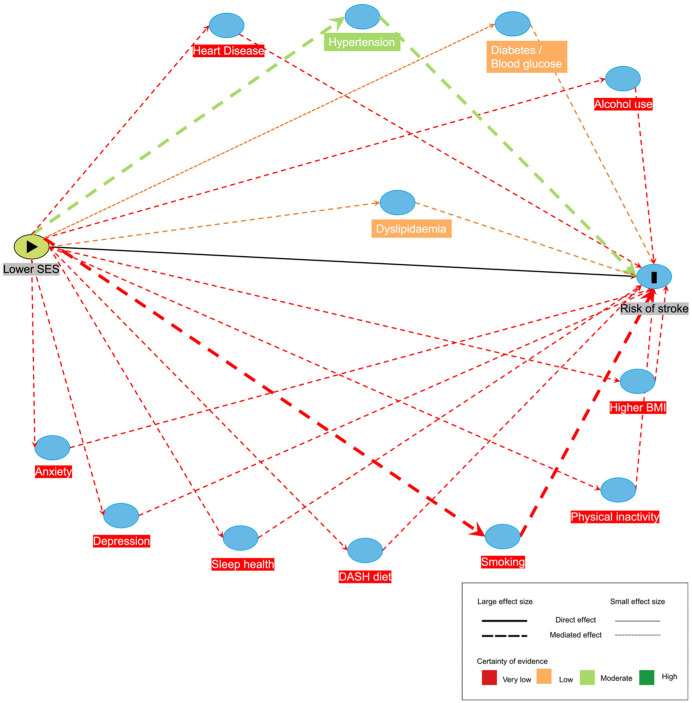
Causal diagrams visualizing hypothesized mediators of the effect of SES on stroke risk. Arrow type denotes direct versus mediated effects; color reflects certainty; and thickness reflects the relative strength of evidence. Relationships between mediators and unmeasured confounding are not depicted. Effect sizes are in Table 2.

Cardiovascular risk factors were the most consistent mediators (Figure 2).^26,31–34,38,40,44^ In UK Biobank (n = 217,013), systolic blood pressure mediated 14–21% of the SES TE on stroke incidence (moderate certainty), BMI 10–11% (low certainty), and smoking 19.9% (very low certainty) (Table 2).^26^ Smaller effects were reported in the Australian Longitudinal Study on Women’s Health (ALSWH; n = 11,468), where smoking mediated 6% for homeownership and 11% for education.^31^ A Greek case–control study and ALSWH found diet and physical inactivity mediated 5–7%.^31,33^ Poor sleep and depression were assessed in three studies and showed small effects (2–13%) with very low certainty, reflecting reliance on self-reported questionnaires^31,33,44^ (Supplemental Table 5).

**Table 2.** Mediation pathways between socioeconomic status and risk of stroke.

Study	Country	SES measure	Mediator	Proportion mediated (95% CI if available)^a^
Carter et al.^26^	UK	Educational attainment	SBP	14.32% (10.65–17.99%)
			BMI	10.35 (7.58–13.12)
			Smoking	19.92% (12.65–27.19%)
			Combined (SBP, BMI, smoking)	41.5% (26.68–56.32%)
Jackson et al.^31^	Australia	Educational attainment/homeownership	Smoking	6.4% / 10.6%
			Alcohol	6% / 9.9%
			Physical inactivity	4.6% / 5.2%
			Depression	10% / 13%
			Hypertension	2.1% / 4.4%
			Diabetes	3.4% / 4.4%
			Heart disease	2.9% / 7.5%
Jeong et al.^32^	South Korea	Income	Metabolic syndrome (SBP, diabetes, dyslipidemia)	21% (⩾2 components) / 27% (⩾3 components)
Lee et al.^34^	South Korea	Cumulative social risk^b^	Framingham risk score^c^	35%
Nandi et al.^38^	USA	Educational attainment	Childhood SES^d^	65%
Kastorini et al.^33^	Greece	Educational attainment	Type of occupation	β = −0.2 (p < 0.001)
			Smoking	β = 0.023 (p = 0.004)
			Physical activity	β = 0.015 (p = 0.02)
		Type of occupation	Smoking	β = −0.04 (p < 0.05)
		Income	Anxiety	β = −1.5 (p < 0.001)
Wang et al.^44^	UK	Educational attainment	Smoking status	17.8% (13.6–22.0%)
			Alcohol intake	3.0% (1.2–4.9%)
			DASH diet	1.8% (0.3–3.2%)
			Sleep health^e^	2.6% (1.0–4.2%)
			Physical activity	2.3% (0.7–3.9%)
			BMI	15.1% (11.1–19.1%)
			Cholesterol	0.7% (–0.2–1.7%)
			Blood glucose	2.3% (0.7–3.9%)
			SBP	21.5% (17.2–25.7%)
			Lifestyle behaviors^f^	27.6% (22.7–45.7%)
			Cardiovascular risk factors^g^	32.9% (27.1–46.6%)

a Proportion mediated indicates the percentage of the total SES effect on stroke outcome explained by the mediator.

b Cumulative social risk: score of low income, low education, and living alone.

c Framingham risk score: age, cholesterol (total and HDL), smoking, diabetes, SBP, and hypertension treatment.

d Childhood SES measured via retrospective reports of parental education, occupation, and household finances before age 16.

e Sleep health: five-point score (chronotype, duration, insomnia, snoring, and sleepiness); ⩾ 4 considered healthy.

f Lifestyle behaviors include current smoking, alcohol use, low DASH diet score, poor sleep, and physical inactivity.

g Cardiovascular risk factors: included SBP, BMI, total cholesterol, and blood glucose.

When combined, behavioral factors (smoking, alcohol, inactivity, sleep, and diet) explained 40–47% of the SES TE in UK Biobank,^26,44^ but longitudinally, this contribution declined across cohorts, driven mainly by falling smoking prevalence.^44^ Joint mediation via SBP, BMI, and blood glucose also fell over time, driven mainly by decreases in blood glucose and SBP, while the proportion mediated by BMI increased.^44^ Metabolic syndrome in South Korea (based on BMI, cholesterol, and SBP) mediated 21% when defined as two or more components and 27% when defined as three or more components and Framingham risk score up to 35%.^32,34^ In the US Health and Retirement Study (HRS), early-life SES showed a strong direct effect, with 65% of the total effect not mediated by adult SES.^38^ The Italian EPICOR cohort found no SES–stroke association, precluding mediation analysis.^40^

### NIE of SES on care, disability, and mortality

**Figure 3.**
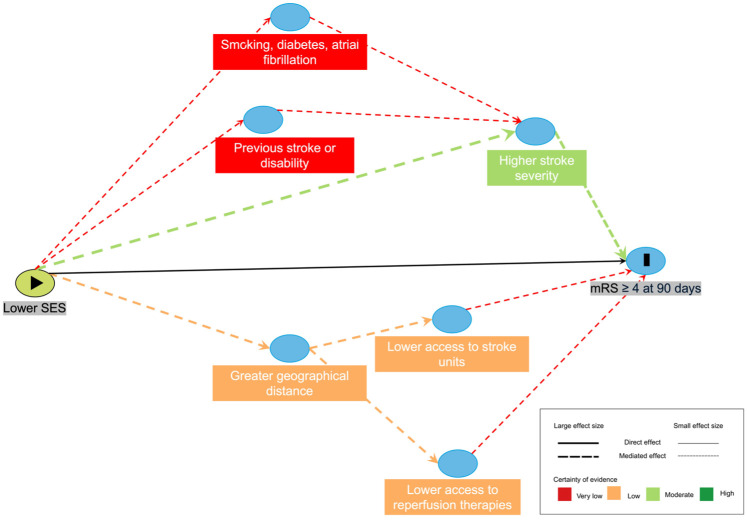
Causal diagrams visualizing hypothesized mediators of the effect of SES on stroke disability at 90 days. Arrow type denotes direct versus mediated effects; color reflects certainty; and thickness reflects the relative strength of evidence. Relationships between mediators and unmeasured confounding are not depicted. Effect sizes are in Table 3.

**Figure 4.**
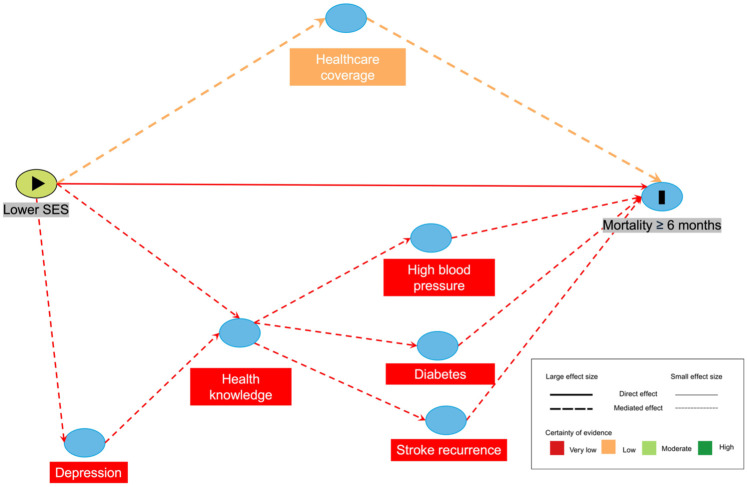
Causal diagrams visualizing hypothesized mediators of the effect of SES on stroke mortality. Arrow type denotes direct versus mediated effects; color reflects certainty; and thickness reflects the relative strength of evidence. Relationships between mediators and unmeasured confounding are not depicted. Effect sizes are in Table 3.

Eleven studies examined mediators of SES inequalities in disability and mortality, mostly focusing on stroke severity,^29,35–37,39,43^ acute care,^27,30,37^ and comorbidities or health behaviors,^28,41,42^ using causal mediation analysis (n = 7),^27–30,35,36,43^ structural equation modeling (n = 3),^39,41,42^ and disparity effects (n = 1).^37^ Outcomes such as mRS and activities of daily living (ADL) are analyzed along a continuum from disability to death (Figures 3 and 4 and Table 3).

**Table 3.** Mediation pathways between socioeconomic status and stroke disability/mortality.

Study	Country	SES measure	Outcome	Time frame	Mediator	Proportion mediated (95% CI if available)^a^
Lindmark et al.^35^	Sweden	Income	Death	3 months	Stroke severity (consciousness level)	Ischemic stroke: 38.5% Hemorrhagic stroke: 57%
Lindmark et al.^36^	Sweden	Educational attainment	Disability and stroke severity	Admission	Risk factors (smoking, diabetes, AF, previous stroke, ADL dependency)	28.5%
					Stroke prevention drugs	−0.6%
Lindmark et al.^37^	Sweden	Education and income	Death or ADL dependency	3 months	Stroke severity	26% (14.9–37%)
					Reperfusion therapy	2.7% (0.5–4.9%)
Potter et al.^39^	USA	Area Deprivation Index	Disability or death (mRS 4–6)	90 days	Stroke severity	94.1%
Ghoneem et al.^29^	USA	Zip code median income	Disability (mRS)	90 days	Infarct volume and NIHSS	64%
Eagles et al.^27^	Canada	Neighborhood deprivation	Access to endovascular thrombectomy	Hospitalization	Distance to center	48%
Hyldgård et al.^30^	Denmark	Income and educational attainment	30-day mortality	30 days	Quality of acute care	0.17% (–0.15% to 0.49%)
			30-day readmission			–0.02% (–0.14% to 0.09%)
Fan and Lam^28^	USA	Educational attainment	Mortality	Up to 20 years	Comorbidities (high blood pressure, diabetes, stroke recurrence)	18%
					Medicaid coverage	24% (high school) / 55% (less than high school)
Zhang et al.^41^	China	Educational attainment	Disability	6 months	Health behaviors	β = −0.23^b^
Zhang et al.^42^	China	Educational attainment	Disability and health behaviors	6 months	Health knowledge	β = 0.218^b^
					Depression	β = −0.270^b^

Note: This table combines disability and mortality outcomes, as many studies use continuous measures (like mRS or ADL) that assess both functional disability and death.

ADL: activities of daily living; AF: atrial fibrillation; BP: blood pressure

a When reported, proportion mediated refers to the percentage of the total effect of SES on stroke outcome explained by the mediator.

b β values represent standardized indirect effects from path analysis models; they reflect the size and direction of mediation pathways from education to outcomes via specific psychosocial or behavioral mediators.

Stroke severity emerged as the strongest mediator of the SES-disability/death association (n = 5).^29,35–37,39^ In Sweden, stroke severity (measured as level of consciousness) explained 38.5% of the income TE on 3-month mortality after ischemic stroke and 57% after hemorrhagic stroke (moderate certainty),^35^ indicating that over one-third of income-related mortality disparities operated through differences in initial stroke severity. A subsequent analysis confirmed stroke severity as the dominant pathway, mediating 26–27% of the TE, while diabetes, hypertension, dyslipidemia, and reperfusion access raised the combined mediation to 40%.^37^ US single-center cohorts reported severity mediating 94% of SES inequalities in 90-day disability or death,^39^ and infarct volume and NIHSS 64% of mRS at 3 months.^29^

Healthcare access and quality showed weaker, context-specific mediations.^27,30,37^ In Canada, distance to stroke centers mediated 48% of inequalities in thrombectomy access, which themselves mediated disability outcomes (low certainty).^27^ By contrast, quality indicators in Denmark and Sweden (timely imaging, thrombolysis, stroke-unit admission, dysphagia screening) mediated little or none of the SES TE on mortality or disability, with estimates ⩽ 2.7% and very low certainty.^30,37^

Comorbidities and health behaviors contributed variably. In Sweden, diabetes, atrial fibrillation, and smoking together mediated 28% of the effect of low education on severity, while prevention medications had negligible effects (low certainty).^36^ In the US HRS, comorbidities mediated 18% and healthcare coverage 22% among individuals with high school education and 55% among those with less than high school education on inequalities in mortality over 20 years (low certainty).^28^ In China, lower education was linked to poorer diet, physical activity, smoking cessation, and adherence, which mediated higher disability at 6 months (very low certainty).^41,42^ In our results, no study assessed secondary care or rehabilitation as mediators.

## Discussion

This systematic review identified mediators of SES-driven inequalities in stroke risk, disability, and mortality. Hypertension emerged as the main driver of risk inequalities (moderate certainty),^26,31,32,34^ and lifestyle (smoking, physical inactivity, alcohol use) and psychological mediators (depression and anxiety) showed low certainty.^31,33,34^ Early SES had a strong NDE on stroke risk.^38^ For post-stroke outcomes, stroke severity at presentation was the dominant mediator of disability inequalities at 3 months (moderate certainty),^29,35–37,39^ indicating that SES inequalities in death/disability are driven by differences in stroke severity at the point of hospital arrival, while health behaviors, comorbidities and access to acute care mediated longer-term mortality inequalities(low or very low certainty).^28,41,42^

Overall, inequalities were largely explained by mediators, rather than direct SES effects.^13,45^ The significant NIE of cardiovascular risk factors is consistent with evidence on SES gradients in these conditions.^20,46–48^ These pathways reflect how adverse living conditions (e.g. access to green spaces, food safety), psychosocial stressors (financial insecurity, occupational strain, discrimination), and environmental exposures (air pollution, housing quality) cluster in lower SES groups and worsen cardiovascular health.^20,46,47,49,50^ Mediation patterns varied by generations,^44^ with smoking declining and diet/obesity increasing in recent generations, highlighting prevention needs tailored to evolving contexts.^44^

Findings align with INTERSTROKE, which attributed 90.7% of global stroke risk to modifiable factors,^51^ yet in this review, these explained less than half of the SES effect, indicating other unmeasured pathways.^48,52^ Early-life SES was only measured in one study (low certainty), yet broader literature indicates that it likely contributes through multimorbidity accumulation.^38,53,54^ Lower early-life SES is associated with earlier onset and faster accumulation of multimorbidity, increasing stroke risk independently of adult SES.^53,54^ Yet no included studies formally examined the NIE linking early-life SES to stroke, highlighting a key evidence gap.

For disability and mortality outcomes, stroke severity at presentation emerged as the dominant mediator, demonstrating that SES inequalities in outcomes are substantially established before patients reach hospital care.^29,35–37,39^ This connects incidence and outcome on a continuum; with lower SES increasing both the likelihood of stroke occurrence and the severity of stroke at presentation via cardiovascular risk factors,^29,35–37,39^ creating a “double burden” whereby disadvantaged groups are both more likely to have a stroke and to have a more severe stroke when it occurs.^29,35–37,39^ Care quality had modest mediating effects, partly due to omission of reperfusion therapies, short follow-up periods, and restriction to HICs with universal healthcare.^27,35–37^ Geographic distance mediated access to reperfusion therapies in Canada, highlighting that care delivery mediates, despite even where upstream factors dominate.^27^

Clinically, hypertension and smoking should remain priority targets for reducing SES inequalities.^26,55–57^ In acute care, improving health-seeking behaviors and access via expanded stroke-unit coverage, reduced delays, and integrated post-stroke follow-up may improve outcomes for low SES patients.^58,59^ Over the longer term, healthcare interventions should include targeted screening for hypertension and smoking and adherence support, complemented by policies addressing behavioral and contextual drivers.^41,42^

Incomplete mediation by cardiovascular risk factors suggests that clinical interventions alone are insufficient to reduce SES inequalities in stroke. Population-level actions (taxation and advertising bans) reduce smoking and, when well designed, can also narrow inequalities,^60,61^ though other approaches (smoke-free policies, mass media campaigns) have shown mixed or even widened inequalities.^62^ The strong influence of early-life SES supports life-course approaches, including school-based prevention^63,64^ and free meal schemes,^63,65^ can modify long-term behaviors but remain difficult to sustain and evaluate.^60,66^

In adulthood, low SES groups are less likely to access routine care,^67,68^ and community-based delivery models are essential. Evidence shows that training lay health workers is more effective when combined with structured community support,^69^ and that social enterprises using cross-subsidies or low-cost models can expand access where universal coverage is lacking.^70^ Improving health literacy and reducing provider bias through culturally adapted communication can also enhance equity.^71^

Systematic recording of SES indicators in health records (such as employment status, housing security, and food access) could support clinicians in recognizing social risk and linking patients to relevant services.^72^ Finally, multisectoral action is essential: housing policies that improve air quality,^72^ access to greenery,^73^ food safety,^74^ and investment in safe walking and cycling infrastructure^75,76^ can simultaneously reduce hypertension, smoking, and care barriers.

## Strengths and limitations

As expected, SES measurement varied across studies, reflecting its dynamic nature and interaction with risk factors over the life course.^13,45^ We addressed this by including only studies using robust causal-mediation methods, conceptualizing SES as an upstream determinant acting through mediating pathways and adopting a life-course perspective.

Our causal diagrams depict mediators reported in the included studies but necessarily simplify inter-relationships that are supported in the wider literature. Diagrams should therefore be read as partial representations of pathways to stroke.

Most studies concentrated on traditional risk factors, with limited attention to emerging pathways such as frailty, discrimination, social support, or stress. Repeated focus on the same mediators risks reinforcing knowledge gaps; future research should expand to underexplored mechanisms.

Findings largely reflect combined stroke types. When reported, mediation differed by subtype, warranting further research.^29,30,35,39^ Most studies were from HICs, limiting generalizability. Study quality was generally low, with GRADE ratings of low/very low certainty and ROBINS-E identifying bias from subjective SES measures and incomplete confounding control.

Measurement error may also have limited validity, particularly for self-reported SES, health behaviors, and psychosocial factors (e.g. depression, anxiety). Many mediators (including cardiovascular risk factors) were assessed only as binary variables, ignoring severity, control, or change over time, likely underestimating their contribution.

Despite these limitations, this systematic review identifies modifiable pathways that offer actionable targets for reducing SES-related stroke inequalities.

## Future research

Significant research gaps remain, particularly in LMIC, where healthcare inequalities may be greater. Further research is needed on rehabilitation access, quality of life, and the long-term impact of early-life SES on stroke severity and recovery.^53^ Greater understanding of how SES intersects with sex, ethnicity, migration, sexual identity, and family status could support more tailored interventions through an intersectional approach.

## Conclusion

Hypertension and smoking constitute the primary mediators through which lower SES increases stroke risk, yet they account for less than half of the TE, indicating that substantial pathways remain unmeasured. Stroke severity represents the strongest mediator underlying the TE of SES on post-stroke disability and mortality at 3 months. In the longer term, SES inequalities in mortality are mediated by hypertension and diabetes, as well as differences in healthcare coverage.

Given the absence of studies from LMICs, future research should prioritize investigations of these mediating pathways in these settings. Additional priorities include better standardization of SES measures, stronger methodological approaches to causal mediation analysis, and exploration of the long-term impact of childhood SES on adult stroke risk. Understanding how geography and rurality affect these NIEs in both high-income and low-income regions remains critical to developing contextualized interventions that target the most influential mediating pathways.

## Supplemental Material

sj-docx-1-wso-10.1177_17474930251399064 – Supplemental material for A systematic review of causal pathways of socioeconomic inequalities in stroke Supplemental material, sj-docx-1-wso-10.1177_17474930251399064 for A systematic review of causal pathways of socioeconomic inequalities in stroke by Camila Pantoja-Ruiz, Lu Liu, Evelyn Lim, Marina Soley-Bori, Wasana Kalansooriya, Eva Emmett, Abdel Douiri, Yanzhong Wang, Ajay Bhalla, Amal R Khanolkar, Divya Parmar, Sabine Landau, Matthew DL O’Connell, C D A Wolfe and Iain J Marshall in International Journal of Stroke
