# Supplementary material for: A systematic review of causal pathways of socioeconomic inequalities in stroke
Source: Int J Stroke. 2025 Nov 12;21(6):801–14. doi: 10.1177/17474930251399064 (PMC13291443; doi:10.1177/17474930251399064)
Supplement: sj-docx-1-wso-10.1177_17474930251399064 – Supplemental material for A systematic review of causal pathways of socioeconomic inequalities in stroke [file sj-docx-1-wso-10.1177_17474930251399064.docx]

**Supplementary material**

[Supplemental Panel 1. PubMed/MEDLINE search strategy for the systematic review 2](#_Toc208395485)

[Supplemental Panel 2. EMBASE search strategy for the systematic review 3](#_Toc208395486)

[Supplemental Panel 3. Inclusion and exclusion criteria 4](#_Toc208395487)

[Inclusion Criteria 4](file:////Users/mariacamilapantojaruiz/Library/Mobile%20Documents/com~apple~CloudDocs/Documents/PhD/2.%20Systematic%20review/Submission%20IJS/IJS%20Revision%20%231/Supplementary.docx#_Toc208395488)

[Exclusion Criteria 4](file:////Users/mariacamilapantojaruiz/Library/Mobile%20Documents/com~apple~CloudDocs/Documents/PhD/2.%20Systematic%20review/Submission%20IJS/IJS%20Revision%20%231/Supplementary.docx#_Toc208395489)

[Supplementary Figure 1. Global Distribution of included articles on mechanisms mediating the effect of SES on stroke risk and outcomes 5](#_Toc208395490)

[Supplementary Table 1. Detailed characteristics of the nineteen articles included in the systematic review. 6](#_Toc208395491)

[Supplementary Table 2: Association Between Low SES and Risk of Stroke 13](#_Toc208395492)

[Supplementary Table 3. Association Between Low SES and Post-Stroke Outcomes 14](#_Toc208395493)

[Supplementary Table 4. ROBINS_E risk of bias assessment across studies evaluating socioeconomic status and stroke outcomes 15](#_Toc208395494)

[Supplementary Table 5. GRADE Assessment of Evidence for Mediating Pathways Between SES and Risk of Stroke 16](#_Toc208395495)

[Supplementary Table 6. GRADE Assessment of Evidence for Mediating Pathways Between SES and Stroke Disability/Mortality 18](#_Toc208395496)

# Supplemental Panel 1. PubMed/MEDLINE search strategy for the systematic review

(("Socioeconomic Factors"[Mesh] OR "Socioeconomic Status"[tiab] OR "Income"[tiab] OR "Education"[tiab] OR "Occupation"[tiab] OR "Health Status Disparities"[tiab])

AND

("Stroke"[Mesh] OR Stroke* OR "Cerebrovascular Accident"[tiab] OR "Cerebral Infarction"[tiab] OR "Brain Ischemia"[Mesh] OR "Ischemic Stroke"[tiab] OR "Hemorrhagic Stroke"[tiab] OR "Cerebrovascular Disorders"[Mesh])

AND

("Causality"[Mesh] OR "Mediation Analysis"[Mesh] OR Mechanisms OR Causal OR Pathways OR “Structural Equation Model*” OR “Mendelian Randomi*” OR “Causal Inference” OR “Path* Analysis” OR “Mediation” OR “mediation analysis” OR "Sensitivity Analyses" OR "Unmeasured Confounding")

AND

("cohort studies"[mesh] OR "case-control studies"[mesh] OR "comparative study"[pt] OR OR "cohort"[tw] OR "compared"[tw] OR "groups"[tw] OR "case control"[tw])

NOT

("Randomized Controlled Trial"[Publication Type] OR "Clinical Trial"[Publication Type] OR "Meta-Analysis"[Publication Type] OR "Systematic Review"[Publication Type] OR "Review"[Publication Type]))

# Supplemental Panel 2. EMBASE search strategy for the systematic review

(TITLE-ABS-KEY("socioeconomic factors" OR "socioeconomic status" OR income OR education OR occupation OR "health status disparities"))

AND

(TITLE-ABS-KEY(stroke* OR "cerebrovascular accident" OR "cerebral infarction" OR "brain ischemia" OR "ischemic stroke" OR "hemorrhagic stroke" OR "cerebrovascular disorders"))

AND

(TITLE-ABS-KEY(causality OR "mediation analysis" OR mechanisms OR causal OR pathways OR "structural equation model*" OR "mendelian randomization" OR "causal inference" OR "path* analysis" OR mediation OR "sensitivity analysis" OR "unmeasured confounding"))

AND

(TITLE-ABS-KEY("cohort studies" OR "case-control studies" OR "comparative study" OR cohort OR compared OR groups OR "case control"))

NOT

(TITLE-ABS-KEY("randomized controlled trial" OR "clinical trial" OR "meta-analysis" OR "systematic review" OR review))

# Supplemental Panel 3. Inclusion and exclusion criteria

**Inclusion Criteria**

- Peer-reviewed original studies analysing the pathways between SES and the incidence, disability or mortality of stroke.
  - SES can be considered as a composite measure or through indicators such as individual or grouped occupation, income, or education.
- Observational study designs: Cohort studies, case-control studies, cross-sectional studies
- Studies employing statistical methods to establish causality, mediation, or influence pathways: Causal inference, structural equation modeling, path analysis
- No restrictions based on sex, gender, age, geographic location, or healthcare settings.
- Studies reporting an estimate of: Hazard ratio (HR), odds ratio (OR), beta coefficients, excess mortality rate ratio (EMRR) with a corresponding 95% confidence interval (CI) or standard error.
- No date restrictions applied; all available literature included.

**Exclusion Criteria**

- Articles that do not differentiate individuals by SES.
- Studies reporting only associations without causal analysis
- Studies without quantification of mediating pathways
- Case reports, editorials, letters, and narrative reviews
- Studies focusing exclusively on pediatric populations
- Animal studies

# Supplementary Figure 1. Global Distribution of included articles on mechanisms mediating the effect of SES on stroke risk and outcomes


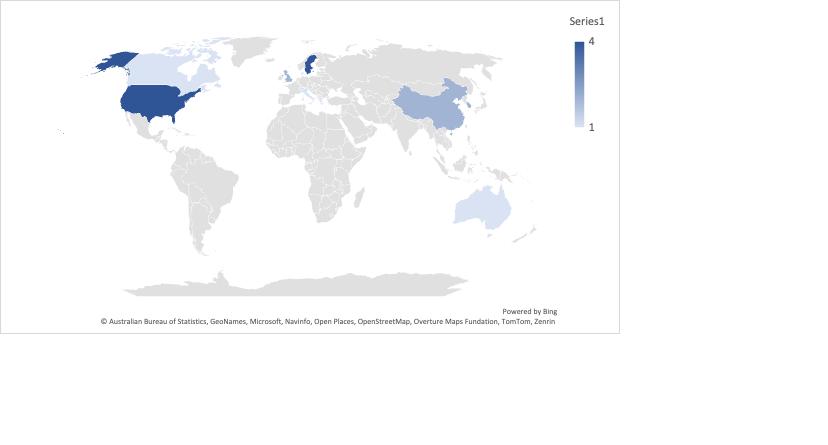


Map showing the number of included studies by country. The United States and Sweden both contributed the highest number (n = 4 each), followed by China, South Korea and the United Kingdom (n = 2 each). Other countries represented include the Canada, Australia, Italy, Greece, and Denmark (n = 1 each).

# Supplementary Table 1. Detailed characteristics of the nineteen articles included in the systematic review.

| **Study ID** | **Author (Year)** | **Country** | **Study Design** | **Sample Size** | **SES Measure(s)** | **Outcome(s)** | **Mediators Examined** | **Mediation Method** | **Key Findings** | **Proportion Mediated*** | **Risk of Bias** |
| --- | --- | --- | --- | --- | --- | --- | --- | --- | --- | --- | --- |
| **Stroke Risk** | | | | | | | | | | | |
| 1 | Carter et al. (2019) | UK | Cohort (prospective)** | 217,013 | Education (highest qualification) | Incidence of first-ever stroke (WHO/ICD) | BMI, systolic blood pressure, smoking behavior | Product of coefficients & difference method | Each additional SD of education (3.6 years) associated with 11% lower risk of stroke (OR 0.89; 95% CI: 0.85 to 0.93) in the observational analysis | BMI: 8% for stroke SBP: 28% for stroke Smoking: 19% for stroke All three: 40% for stroke | Some concerns |
| 2 | Jackson et al. (2013) | Australia | Cohort (prospective) | 11,468 | Education, homeownership | Incidence of first-ever stroke (self-report/registry linkage) | Lifestyle factors, biological factors, psychosocial factors | Percentage attenuation in β coefficients | Women with lowest education had 2.45× higher odds of stroke; non-homeowners had 1.63× higher odds | Individual attenuation effects:  Smoking: 6.4%–10.6%  Alcohol: 6%–9.9%  Physical inactivity: 4.6%–5.2%  Depression: 10%–13%  Hypertension: 2.1%–4%  Diabetes: 3.4%–4.4%  Heart disease: 2.9%–7.5% | High |
| 3 | Jeong et al. (2022) | South Korea | Cohort (prospective) | 213,526 | Income (insurance premium) | Incidence of stroke (ICD codes) | Metabolic syndrome diseases (hypertension, diabetes, dyslipidemia) | Causal mediation with weighting approach | Lower income associated with higher stroke risk and higher risk of metabolic syndrome diseases | Hypertension, diabetes, and dyslipidaemia jointly as a composite mediator mediated 26.6% of the income effect in stroke risk | High |
| 4 | Lee et al. (2020) | South Korea | Cross-sectional | 19,147 | Cumulative social risk score (income, education, living alone) | Stroke, MI, angina - Odds of physician-diagnosed stroke (self-report) | Framingham Risk Score components | Product-of-coefficient methods | Each 1-unit increase in social risk associated with 89.4% higher stroke risk controlling for FRS | For stroke: 35% mediated by FRS (65% not mediated) | High |
| 5 | Nandi et al. (2012) | USA | Cohort (longitudinal, HRS panel) | 9,055 (stroke-free at baseline) | Early-life SES (parental education, occupation, region) | Self-reported physician-diagnosed stroke (survey waves) | Adult SES | Marginal structural models | Early-life SES had significant direct effects on stroke not mediated by adult SES | 65% of the effect of early-life SES on stroke risk was not mediated by adult SES, indicating a strong direct pathway | High |
| 6 | Kastorini et al. (2015) | Greece | Case–control (hospital-based) | 1,000 | Education, financial satisfaction, occupation | Odds of first-ever ischaemic stroke and ACS | Hypertension, hypercholesterolemia, diabetes, lifestyle factors | Path analysis (structural equation modeling) | Education affected occupation, financial satisfaction, and lifestyle factors; different pathways for ACS vs. stroke | Indirect effects not quantified as proportion mediated. Reported standardised β coefficients:  Education → Financial satisfaction: β = 0.25  Financial satisfaction → Anxiety: β = 0.35  Anxiety → Lifestyle: β = 0.22  Lifestyle → Stroke: β = 0.31  Anxiety → Stroke: β = 0.15 | Some concerns |
| 7 | Ricceri et al. (2016) | Italy | Cohort (prospective, EPICOR) | 43,791 | Educational level | Cardiovascular events, cerebrovascular events | Clinical risk factors, lifestyle factors | Cox regression and Structural Equation Modeling | Lower education associated with increased CE risk; no significant association with CBVD | Indirect effects not quantified. Lifestyle factors explained more of the SES–cardiovascular risk association than clinical factors, but SES association with stroke was not significant (HR 1.7; 0.85-1.38) therefore no SEM was conducted. | Some concerns |
| 8 | Wang et al. (2025) | UK | Cohort (prospective, UK Biobank) | 447,227 | Educational attainment | Stroke risk | Healthy lifestyle (smoking, alcohol, diet, sleep, physical activity), metabolic risk (SBP, BMI, glucose, cholesterol) | Counterfactual-based causal mediation | Education-related stroke risk was partly mediated by lifestyle and metabolic factors. Strongest individual mediators were SBP (21.5%), smoking (17.8%), and BMI (15.1%). | Lifestyle: 27.6% (95% CI: 22.7–45.7); Metabolic: 32.9% (95% CI: 27.1–46.6) | Moderate |
| **Disability after stroke** | | | | | | | | | | | |
| 9 | Eagles et al. (2023) | Canada | Cohort (retrospective) | 1,335 | Area-based deprivation (Pampalon index) | Disability (Endovascular Therapy (EVT) access; distance to centre) | Distance to nearest comprehensive stroke center | Counterfactual-based causal mediation analysis using the mediation R package (logistic models for mediator and outcome; 500 bootstrap replications). | Patients from most deprived neighborhoods less likely to receive EVT (OR 0.43); living farther from comprehensive stroke centers | 48% of effect of neighborhood deprivation on EVT receipt mediated by distance to CSC | High |
| 10 | Ghoneem et al. (2022) | USA | Cohort (prospective) | 1,098 | Zip code median income, area deprivation index | 90-day post-stroke disability (mRS) | Infarct volume (DW-MRI), NIHSS score | Formal mediation (single & dual mediator models) | Lower SES independently associated with larger infarct volumes and higher NIHSS scores | An estimated 64% of the SES-disability association was attributed to the indirect pathway through stroke severity (infarct volume and NIHSS), calculated as indirect effect divided by total effect. | High |
| 11 | Lindmark et al. (2022) | Sweden | Cohort (retrospective) | 86,316 | Education | Stroke severity | Risk factors, stroke prevention drugs | Causal inference with interventional disparity effects | Low education associated with excess risk of severe stroke (risk difference 1.4%) | 28.5% mediated by risk factors (primarily diabetes, previous stroke, ADL dependency) Stroke prevention drugs: negligible (-0.6%) | High |
| 12 | Lindmark et al. (2024) | Sweden | Cohort (register-based) | 6,910 | Composite (education + income) | ADL at 3 months | Smoking, metabolic health (diabetes, hypertension, statin use), atrial fibrillation, stroke severity | Causal mediation analysis (simulation-based interventional effects) | Intervening on smoking and metabolic health could reduce SES disparities in PROMs by 14–45%. | Stroke severity and consciousness at presentation mediated 24.5% of SES differences in ADL dependency. | Some concerns |
| 13 | Zhang et al. (2020) | China | Cohort (nested RCT) | 151 | Education | Health behavior, BP control, disability (mRS) | Physical activity, diet, smoking, medication adherence | Structural Equation Modeling | Higher education predicted greater stroke knowledge and health beliefs, improving health behavior | Health knowledge and beliefs mediated behavior change: β = 0.186 (indirect), β = 0.391 (direct). Indicates partial mediation of education effects on stroke-related behaviors | High |
| 14 | Zhang et al. (2022) | China | Cross-sectional | 121 | Education | Health behavior 6 months post-stroke | Depression, chronic illness resources, health knowledge | Structural Equation Modeling | Chronic illness resources had largest effect on behavior; low education, male sex, and depression associated with poorer behavior | Health knowledge had a total effect on behaviour of β = 0.436, with approximately half mediated via depression and education. The direct effect of education on behaviour was β = 0.168. | Some concerns |
| **Post-stroke mortality** | | | | | | | | | | | |
| 15 | Lindmark et al. (2023) | Sweden | Cohort (nationwide) | 25,846 | Composite of education and income | Death or ADL dependency at 3 months | Comorbidities, stroke severity, acute care (reperfusion, stroke unit) | Causal mediation with interventional disparity effects | Low SES associated with 5.4% increased risk vs. mid SES and 10.1% vs. high SES | ~40% of excess risk could be eliminated by equalizing mediators; stroke severity most important (26-27%) | Some concerns |
| 16 | Lindmark et al. (2020) | Sweden | Cohort (nationwide) | 57,936 | Income | 3-month case fatality | Stroke severity (level of consciousness) | Causal mediation analysis | Patients in lowest income tertile had 3.2% increased absolute risk of death after ICH and 1.0% after ischemic stroke | ICH: 57% mediated by stroke severity Ischemic stroke: 38.5% mediated by stroke severity | High |
| 17 | Hyldgård et al. (2023) | Denmark | Cohort (retrospective) | 59,066 | Education and income | 30-day mortality, 30-day readmission | Quality of early stroke care (5 performance measures) | Formal mediation (Lange method) | Income-related inequality in 30-day mortality unchanged 2003-2018; quality of care did not mediate | Proportion mediated 0.17% (-0.15% to 0.49%) | High |
| 18 | Fan & Lam (2021) | USA | Cohort (longitudinal) | 17,228 (MI) 14,113 (stroke) person-years | Educational attainment | All-cause mortality after MI or stroke | Spousal education, wealth, health insurance, health behaviors, comorbidities | Karlson–Holm–Breen (KHB) method | Educational gradient found in mortality after MI and stroke; stronger for more recent cohorts | For stroke: occupation (42-60%), wealth (41-73%), Medicaid coverage (24-55%), comorbidities (18%)† | High |
| 19 | Potter et al. (2023) | USA | Cohort (retrospective) | 677 | Area Deprivation Index | 90-day severe disability/death (mRS 4-6) | Stroke severity, cerebral small vessel disease | Structural Equation Modeling (Baron & Kenny) | Stroke severity completely mediated relationship between deprivation and poor outcomes | Stroke severity mediated 94.1% of effect; CSVD mediated 4.9% (non-significant) | Some concerns |

**Abbreviations:** ADL: Activities of Daily Living; ACS: Acute Coronary Syndrome; BMI: Body Mass Index; BP: Blood Pressure; CBVD: Cerebrovascular Disease; CE: Cardiovascular Events; CIRS: Chronic Illness Resource Survey; CRS-HBM: Cognitive-Response Strategy based on Health Belief Model; CSC: Comprehensive Stroke Center; CSVD: Cerebral Small Vessel Disease; EVT: Endovascular Thrombectomy; FRS: Framingham Risk Score; ICH: Intracerebral Hemorrhage; MI: Myocardial Infarction; MR: Mendelian Randomization; mRS: modified Rankin Scale; NIHSS: National Institutes of Health Stroke Scale; OR: Odds Ratio; RCT: Randomized Controlled Trial; SBP: Systolic Blood Pressure; SD: Standard Deviation; SES: Socioeconomic Status

* When reported, proportion mediated refers to the percentage of the total effect of SES on stroke outcome explained by the mediator.

** Also includes Mendelian Randomization analysis; only observational results reported here

† KHB (Karlson–Holm–Breen) method decomposes total effects into direct and indirect components in non-linear models. Reported values indicate proportion of the total effect explained by each mediator.

# Supplementary Table 2: Association Between Low SES and Risk of Stroke

| **Study** | **Country** | **Study Design** | **Sample Size** | **SES Measure** | **Effect Size (95% CI)** |
| --- | --- | --- | --- | --- | --- |
| Carter et al. (2019) | UK | Observational analysis | 217,013 | Educational attainment | OR 1.12 (1.07-1.17) per 3.6 of additional education |
| Jackson et al. (2013) | Australia | Cohort | 11,468 women | Education (lowest vs. highest) | OR 2.57 (1.42–4.65) |
| Jackson et al. (2013) | Australia | Cohort | 11,468 women | Homeownership (no vs. yes) | OR 1.99 (1.39–2.84) |
| Jeong et al. (2022) | South Korea | Cohort | 213,526 | Income (Medical Aid vs. high) | HR 1.19 (1.10-1.29 |
| Lee et al. (2020) | South Korea | Cross-sectional | 19,147 | Cumulative social risk score | OR 1.89 (95% CI 1.64-2.17)per 1-unit increase |
| Nandi et al. (2012) | USA | Cohort | 9,055 | Early-life SES (lowest vs. highest quartile) | RR 1.49 (1.11–2.00) |
| Ricceri et al. (2016) | Italy | Cohort | 43,791 | Educational attainment | HR 1.08 (0.85-1.38) |
| Wang et al. (2025) | UK | Cohort | 447,227 | Educational attainment | OR 1.12 (1.07-1.17) per 3.6 of additional education |

# Supplementary Table 3. Association Between Low SES and Post-Stroke Outcomes

| **Study** | **Country** | **Study Design** | **Sample Size** | **SES Measure** | **Outcome** | **Time Frame** | **Effect Size (95% CI)** |
| --- | --- | --- | --- | --- | --- | --- | --- |
| Ghoneem et al. (2022) | USA | Prospective cohort | 1,098 | Zip code median income | Disability (mRS) | ~90 days | β = 0.074-SD increase in infarct volume per 1-SD decrease in income |
| Hyldgård et al. (2023) | Denmark | Retrospective cohort | 59,066 | Income (low vs. high) | Mortality | 30 days | RR 1.44 (1.12-1.88) in 2015-18 |
| Lindmark et al. (2020) | Sweden | Cohort | 57,936 | Income (lowest tertile vs. higher) | Mortality | 3 months | Absolute risk difference: 3.2% for ICH, 1.0% for ischemic stroke |
| Lindmark et al. (2022) | Sweden | Retrospective cohort | 86,316 | Education (primary only vs. higher) | Stroke severity | At admission | Absolute risk difference: 1.4% (1.0%-1.8%) for severe stroke |
| Lindmark et al. (2023) | Sweden | Nationwide cohort | 25,846 | Composite (education and income) | Death or ADL dependency | 3 months | Absolute risk difference: 5.4% (low vs. mid SES) 10.1% (low vs. high SES) |
| Lindmark et al. (2024) | Sweden | Register-based cohort | 6,910 | Composite of education and income | ADL dependency | 3 months | Absolute risk difference: 6.5% (low vs mid SES); 8.4% (low vs high SES) |
| Potter et al. (2023) | USA | Retrospective cohort | 677 | Area Deprivation Index | Severe disability or death (mRS 4-6) | 90 days | OR 2.18 (1.45-3.26) |

**Abbreviations:** mRS = modified Rankin Scale; ICH = Intracerebral Hemorrhage; ADL = Activities of Daily Living; BP = Blood Pressure; aOR = adjusted Odds Ratio; RR = Risk Ratio; HR = Hazard Ratio

# Supplementary Table 4. ROBINS_E risk of bias assessment across studies evaluating socioeconomic status and stroke outcomes

| **Study** | **D1** | **D2** | **D3** | **D4** | **D5** | **D6** | **D7** | **Overall** |
| --- | --- | --- | --- | --- | --- | --- | --- | --- |
| **Carter2019** | Low | Some concerns | Some concerns | Low | Low | Some concerns | Low | Some concerns |
| **Eagles2023** | Low | Some concerns | Low | Some concerns | Some concerns | Some concerns | Some concerns | High |
| **Fan2021** | Low | Some concerns | Low | Some concerns | Some concerns | Some concerns | Low | High |
| **Ghoneem2019** | Low | Low | Some concerns | Some concerns | Some concerns | Low | Low | High |
| **Hyldgård2022** | Low | Low | Some concerns | Some concerns | Some concerns | Some concerns | Low | High |
| **Jackson2014** | Low | Some concerns | Some concerns | Some concerns | Some concerns | Some concerns | Low | High |
| **Jeong2022** | Low | Low | Some concerns | Some concerns | Some concerns | Low | Low | High |
| **Kastorini2015** | Low | Some concerns | Some concerns | Low | Low | Low | Low | Some concerns |
| **Lee2020** | Low | High | Some concerns | Low | Low | Low | Low | High |
| **Lindmark2020** | Some concerns | Some concerns | Some concerns | Low | Some concerns | Low | Low | High |
| **Lindmark2022** | Some concerns | Some concerns | Some concerns | Low | Some concerns | Low | Low | High |
| **Lindmark2023** | Low | Low | Low | Low | Some concerns | Low | Low | Some concerns |
| **Lindmark2024** | Some concerns | Some concerns | Low | Low | Some concerns | Some concerns | Low | Some concerns |
| **Nandi2012** | Low | High | Some concerns | Low | Some concerns | Low | Low | High |
| **Potter2023** | Low | Low | Low | Some concerns | Some concerns | Low | Low | Some concerns |
| **Ricceri2016** | Low | Low | Low | Low | Some concerns | Low | Low | Some concerns |
| **Wang2025** | Low | Some concerns | Some concerns | Low | Some concerns | Low | Low | Some concerns |
| **Zhang2020** | Low | Low | Low | Some concerns | Some concerns | Low | Low | High |
| **Zhang2022** | Low | Low | Low | Low | Some concerns | Low | Low | Some concerns |

ROBINS-E: Risk Of Bias in Non-randomised Studies - of Exposures. The tool evaluates seven domains: (D1) Bias due to confounding, (D2) Bias from measurement of the exposure, (D3) Bias in selection of participants into the study/analysis, (D4) Bias due to post-exposure interventions, (D5) Bias due to missing data, (D6) Bias from measurement of the outcome, and (D7) Bias in selection of the reported result. Overall risk was rated as low, moderate, serious, or critical.

# Supplementary Table 5. GRADE Assessment of Evidence for Mediating Pathways Between SES and Risk of Stroke

| **Mediator** | **# of studies** | **Study design** | **Risk of bias** | **Inconsistency** | **Indirectness** | **Imprecision** | **Other considerations** | **Certainty** |
| --- | --- | --- | --- | --- | --- | --- | --- | --- |
| Hypertension / blood pressure | 5 | Cohort (5) | Serious (–1) | Not serious | Not serious | Not serious | Large magnitude of effect (+1) | ⨁⨁⨁◯ MODERATE |
| Body mass index / obesity | 3 | Cohort (3) | Serious (–1) | Not serious | Not serious | Serious (–1) | None | ⨁⨁◯◯ LOW |
| Diabetes | 4 | Cohort (4) | Serious (–1) | Not serious | Not serious | Serious (–1) | None | ⨁⨁◯◯ LOW |
| Dyslipidaemia | 3 | Cohort (3) | Serious (–1) | Not serious | Not serious | Serious (–1) | None | ⨁⨁◯◯ LOW |
| Metabolic syndrome components† | 2 | Cohort (2) | Serious (–1) | Not serious | Not serious | Serious (–1) | Large magnitude of effect (+1) | ⨁⨁◯◯ LOW |
| Smoking | 4 | Cohort (3), Case-control (1) | Serious (–1) | Serious (–1) | Not serious | Not serious | None | ⨁◯◯◯ VERY LOW |
| Alcohol consumption | 2 | Cohort (2) | Serious (–1) | Not serious | Not serious | Serious (–1) | None | ⨁◯◯◯ VERY LOW |
| Physical activity | 3 | Cohort (2), Case-control (1) | Serious (–1) | Not serious | Not serious | Serious (–1) | None | ⨁◯◯◯ VERY LOW |
| DASH diet | 1 | Cohort (1) | Serious (–1) | Not assessed | Not serious | Serious (–1) | None | ⨁◯◯◯ VERY LOW |
| Sleep health | 1 | Cohort (1) | Serious (–1) | Not assessed | Not serious | Serious (–1) | None | ⨁◯◯◯ VERY LOW |
| Depression / anxiety | 2 | Cohort (1), Case-control (1) | Serious (–1) | Not serious | Serious (–1) | Serious (–1) | None | ⨁◯◯◯ VERY LOW |
| Early-life SES (direct effect‡) | 1 | Cohort (1) | Serious (–1) | Not assessed | Not serious | Serious (–1) | Large magnitude of effect (+1) | ⨁⨁◯◯ LOW |

**† Metabolic syndrome components refer to the combined mediation of systolic blood pressure, BMI, blood glucose, and cholesterol.**

**‡ Early-life SES refers to the proportion of the SES–stroke relationship not mediated by adult SES, based on retrospective parental education, occupation, and financial circumstances.**

**Explanatory notes:** This assessment represents an adaptation of the GRADE approach for mediation analysis, as standard GRADE methodology was designed for intervention studies rather than mediation pathways.

- **Risk of bias**: Based on ROBINS-E scores, most studies had some concerns, with few rated low or high risk.
- **Inconsistency**: Rated as serious for smoking due to varying estimates of proportion mediated (6.4%-19% across studies) and differences in how smoking was measured.
- **Indirectness**: Rated as serious for depression/anxiety as these were often measured as secondary factors rather than primary mediators of interest.
- **Imprecision**: Rated as serious when confidence intervals were wide or not reported, or when based on small sample sizes or single studies.
- **Other considerations**: Large magnitude of effect (+1) was applied when the mediator consistently explained >20% of the SES-stroke relationship across multiple studies (hypertension) or when a single study showed substantial mediation (>40%). This represents an adaptation of the GRADE approach which considers large relative risks (e.g., RR >2 or <0.5) or very large relative risks (RR >5 or <0.2) for intervention effects.

# Supplementary Table 6. GRADE Assessment of Evidence for Mediating Pathways Between SES and Stroke Disability/Mortality

| **Mediator** | **# of studies** | **Study design** | **Risk of bias** | **Inconsistency** | **Indirectness** | **Imprecision** | **Other considerations** | **Certainty** |
| --- | --- | --- | --- | --- | --- | --- | --- | --- |
| **Stroke severity at presentation** | 5 | Cohort (5) | Serious (-1) | Not serious | Not serious | Not serious | Large magnitude of effect (+1), Consistency across studies (+1) | ⨁⨁⨁◯ MODERATE |
| **Distance to stroke centres/geographical access** | 1 | Cohort | Serious (-1) | Not assessed (single study) | Not serious | Serious (-1) | Large magnitude of effect (+1) | ⨁⨁◯◯ LOW |
| **Reperfusion therapies/stroke unit access** | 2 | Cohort (2) | Serious (-1) | Serious (-1) | Not serious | Serious (-1) | None | ⨁◯◯◯ VERY LOW |
| **Quality of acute stroke care** | 1 | Cohort | Serious (-1) | Not assessed (single study) | Not serious | Serious (-1) | None | ⨁◯◯◯ VERY LOW |
| **Cardiovascular comorbidities** | 3 | Cohort (3) | Serious (-1) | Not serious | Not serious | Serious (-1) | None | ⨁⨁◯◯ LOW |
| **Health behaviours** | 2 | Cohort (2) | Serious (-1) | Not serious | Serious (-1) | Serious (-1) | None | ⨁◯◯◯ VERY LOW |
| **Healthcare coverage** | 1 | Cohort | Serious (-1) | Not assessed (single study) | Not serious | Serious (-1) | Large magnitude of effect (+1) | ⨁⨁◯◯ LOW |
| **Depression** | 1 | Cohort | Serious (-1) | Not assessed (single study) | Serious (-1) | Serious (-1) | None | ⨁◯◯◯ VERY LOW |

**Explanatory notes:**

- This assessment represents an adaptation of the GRADE approach for mediation analysis, as standard GRADE methodology was designed for intervention studies rather than mediation pathways.
- **Risk of bias**: ROBINS_E Score
- **Inconsistency**: Rated as serious for reperfusion therapies/stroke unit access due to conflicting findings between studies (Swedish study showed modest mediation while Danish study found no mediating effect).
- **Indirectness**: Rated as serious for health behaviours and depression as these were measured as mediators in pathways to intermediate outcomes (e.g., blood pressure control) rather than directly to final disability/mortality outcomes.
- **Imprecision**: Rated as serious when confidence intervals were wide or not reported, or when based on small sample sizes or single studies.
- **Other considerations**: Large magnitude of effect (+1) was applied when the mediator consistently explained >20% of the SES-stroke relationship across multiple studies (hypertension) or when a single study showed substantial mediation (>40%). This represents an adaptation of the GRADE approach which considers large relative risks (e.g., RR >2 or <0.5) or very large relative risks (RR >5 or <0.2) for intervention effects. Consistency across healthcare systems (+1) was applied to stroke severity as the finding was replicated across diverse healthcare systems (US, Sweden, Denmark).
